# Green-Emitting Gd_3_Ga_5_O_12_: Tb^3+^ Nanoparticles Phosphor: Synthesis, Structure, and Luminescence

**DOI:** 10.1186/s11671-017-2032-x

**Published:** 2017-04-07

**Authors:** A. Luchechko, L. Kostyk, S. Varvarenko, O. Tsvetkova, O. Kravets

**Affiliations:** 1 Department of Sensor and Semiconductor Electronics, Ivan Franko National University of Lviv, Tarnavskogo St. 107, 79017 Lviv, Ukraine; 2 Department of Organic Chemistry, Lviv Polytechnic National University, Bandera St. 12, 79013 Lviv, Ukraine

**Keywords:** Gd_3_Ga_5_O_12_ garnet, Tb^3+^ ions, Nano- and microceramics, Nanoparticles, Co-precipitation method, Polyethylene glycol (PEG), Excitation spectra, X-ray luminescence, Photoluminescence

## Abstract

Nano- and microceramics of Gd_3_Ga_5_O_12_ garnet doped with 1 mol % Tb^3+^ ions were synthesized via co-precipitation and high-temperature solid-state reaction methods. X-ray diffraction measurements confirmed the formation of the garnet structure with *Ia3d* space group in all investigated samples. Atomic force microscopy surface images and grain-size distribution diagrams of Gd_3_Ga_5_O_12_: 1 mol % Tb^3+^ nanoceramics with 300 and 400 g/mol of polyethylene glycol (PEG) were obtained. The relationship between the content of polyethylene glycol and the particle size of Gd_3_Ga_5_O_12_: Tb^3+^ phosphors was revealed. An intense broad band (*λ*_m_ = 266 nm) related to spin-allowed 4*f* ^8^-4*f* ^7^5*d* ^1^ transitions of Tb^3+^ ions was found in photoluminescence excitation spectra of Gd_3_Ga_5_O_12_: Tb^3+^ nanocrystalline ceramics with PEG-300 and PEG-400 at 300 K. The broad excitation band caused by spin-forbidden (*λ*_m_ = 295 nm) 4*f*-5*d* transitions in Tb^3+^ ions was additionally observed in the photoluminescence excitation spectra of Gd_3_Ga_5_O_12_: Tb^3+^ microceramics. Emission of Tb^3+^ ions under X-ray and UV excitations is presented by two groups of sharp lines which correspond to ^5^D_3_ and ^5^D_4_ → ^7^F_j_ transitions of Tb^3+^ ions with the most intense line at 546 nm (^5^D_4_ → ^7^F_5_). It was established that the increasing of PEG content leads to the decreasing of the X-ray and photoluminescence emission intensities.

## Background

Garnet materials, both in the bulk and in the nanocrystalline form, have found numerous applications and therefore have great interest for researchers [1]. Gadolinium gallium garnet (Gd_3_Ga_5_O_12_) doped with bismuth is a perspective material owing to its potential applications in the development of blue phosphors, X-ray, and cathodoluminescence screens and scintillators [2, 3]. Furthermore, gadolinium gallium garnet is an important material suitable as a host for luminescent trivalent lanthanide and transition metal ions [4]. Rare earth-doped gadolinium gallium garnet has attracted much attention as an important material for many applications in optoelectronics, laser physics, biomedicine, and other areas [1, 4–6]. The Tb^3+^ are attractive emitting ions because of high quantum efficiency related to the large energy gap between the emitting states and the low lying ^7^F_J_ (*J* = 0, 1, …, 6) ground states that, apart from Gd^3+^, are the largest within lanthanides [4].

In the last years, nanocrystalline oxide materials have become extremely popular and have gained increasing technological importance. Particular attention has been devoted to the investigation of nano-sized oxide particles that can emit light in an efficient and controlled way [1]. Developing of the fabrication technology of high-quality nanocrystalline powder and ceramics materials also has great interest since the optical properties of nanocrystals strongly depend on their size and conditions of syntheses [5, 6].

The polycrystalline and nanocrystalline ceramic oxide materials, especially with garnet structure, doped with trivalent rare earth, can exceed the single-crystal analogs in some physical properties, such as larger doping concentrations with controllable distribution in the volume of material, elevated compositional versatility, and increased mechanical and thermal properties [5–7]. Moreover, the technology of their preparation is more economical compared with the standard crystal grown processes.

This paper presents the structural and luminescent properties of Tb^3+^-doped Gd_3_Ga_5_O_12_ garnets prepared by the co-precipitation method using the aqueous ammonia as the precipitant and polyethylene glycol as the polymeric agent, which change agglomeration of the nanoparticles and facilitation of the surface modification. Comparative analysis of luminescence characteristics of prepared micro- and nanoceramics also was done.

## Methods

**Fig. 1:**
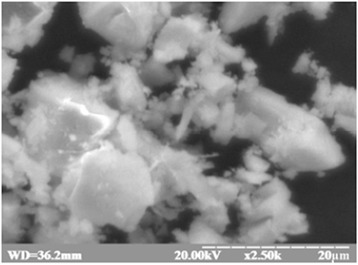
Image of the Gd_3_Ga_5_O_12_ precipitate obtained by co-precipitation method

Nanopowders of Tb^3+^-doped Gd_3_Ga_5_O_12_ garnet were prepared by the co-precipitation method in a polyethylene glycol (PEG)-assisted process. In this method, product components precipitate as insoluble salts or hydroxides. Briefly, appropriate stoichiometric quantities of β-Ga_2_O_3_, Gd_2_O_3_ were used as starting materials. All components were at least 4-N grade of purity. Doping was provided with 1 mol % of Tb_4_O_7_. The mix of oxides was dissolved in 15% nitric acid (4 N) through heating to 100 °C. Then a suitable amount of PEG (molecular weight 300, 400 g/mol) was added to the solution. A chemical reaction took place between the obtained nitrates and the polyethylene glycol. After complete dissolving of starting materials and continuous mixing, the mixture was cooled to 0 ± 2 °C with the slow addition of precipitant—8% of ammonia (NH_4_OH). The precursor was continuously added during mixing in the magnetic mixer till pH of the solution stabilized at 10–11 to ensure small dispersion and homogeneity of deposition. Obtained material was multiple times washed with distilled water till alkaline environment was reached (pH = 7). The final step, separation with Shott’s filter and drying in the vacuum desiccator with humidity absorbent (P_2_O_5_) were done. Further drying of the material was held at 40 °C for 12 h in air. Obtained precipitate (Fig. 1) was milled in an agate mortar and annealed at different temperatures (850 °C) for 4 h in the air. Finally, the sintered nanopowders were milled once again in an agate mortar. Nanoceramic pellets (Nano PEG-300, Nano PEG-400) were formed by uniaxially pressing the powders in a steel die (8 mm in diameter) at 150 kg/cm^2^. Obtained samples were annealed at 1000 °C. Some more details of nanoceramics synthesis are also shown in [8].

Microceramic samples of Gd_3_Ga_5_O_12_ doped with 1 mol % Tb^3+^ were prepared by high-temperature solid-state reaction method. Gadolinium oxide (Gd_2_O_3_), β-gallium oxide (β-Ga_2_O_3_), and terbium oxide (Tb_4_O_7_) were used as the starting materials. All reagents were at least 4-N grade of purity. Obtained tablets were pressed and then annealed at 1200 °C for 8 h in the air.

The morphology of nanoceramics were obtained with an atomic force microscope (ASM) Solver P47H-PRO. Surface morphology investigations of Gd_3_Ga_5_O_12_ microceramic samples of were performed with scanning electron microscope REMMA-102-02.

X-ray diffraction (XRD) analyses were performed on STOE STADI P diffractometer with linear position-sensitive detector (PSD) using an X-ray tube with Cu anode (Kα_1_-radiation, *λ =* 1.5406 Å) in the “Interfaculty scientific-educational laboratory of X-ray structure analysis” of Ivan Franko National University of Lviv. The step of scanning was equal 0.005°. Analysis of diffraction peaks was realized with STOE WinXPOW software package.

Home-made setup based on an SF-4A quartz monochromator was used to investigate the X-ray luminescence. X-ray excitation was performed by a microfocus X-ray tube (45 kV, 0.3 mA) with a copper anticathode through a beryllium window mounted on the cryostat.

Photoluminescence measurements were carried out in the 220-820 nm spectral range on spectrofluorometer CM2203. All excitation and luminescence spectra were obtained with the spectral resolution of 0.5 nm and automatically corrected by lamp intensity and photomultiplier tube sensitivity. Excitation of luminescence was performed with 150-W Xenon lamp. A Hamamatsu R928 photomultiplier was used as the luminescent detector. The luminescence studies were carried out at room temperature.

## Results and Discussion

**Fig. 2:**
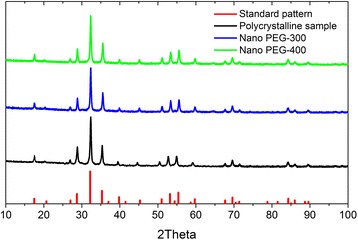
X-ray diffraction patterns of Gd_3_Ga_5_O_12_: Tb^3+^ synthesized via conventional solid-state reaction technique (*black line*) and co-precipitation method with 300 and 400 g/mol of PEG (*blue* and *green lines*, respectively). Vertical *red lines* represent standard diffraction pattern (JCPDS Card No. 13-0493)

Figure 2 represents X-ray diffraction (XRD) patterns of gadolinium gallium garnet samples obtained with different techniques. Microcrystalline ceramics of Gd_3_Ga_5_O_12_: Tb^3+^, as well as nanocrystalline ceramics (Nano PEG-300, Nano PEG-400), show diffraction peaks with respect to Standard diffraction pattern JCPDS Card No. 13-0493. It confirms the formation of a garnet structure with a cubic space group *O*_*h*_^*10*^ (*Ia3d*). No additional phases were detected.

The crystallites size estimation was provided via calculation with Scherrer’s equation for 32.35° diffraction peak:

$$ D = 0.9\lambda /\beta\;cos\;\theta $$

Here *D* is the average grain size; *λ* is the wavelength of X-rays equal to 0.15406 nm; *θ* is the diffraction angle; and β is the full width at the half maximum of the main diffraction peak. Thus, the calculated average crystalline grain sizes are 43.7 and 38.9 nm for Nano PEG-300 and Nano PEG-400 ceramics, respectively.

**Fig. 3:**
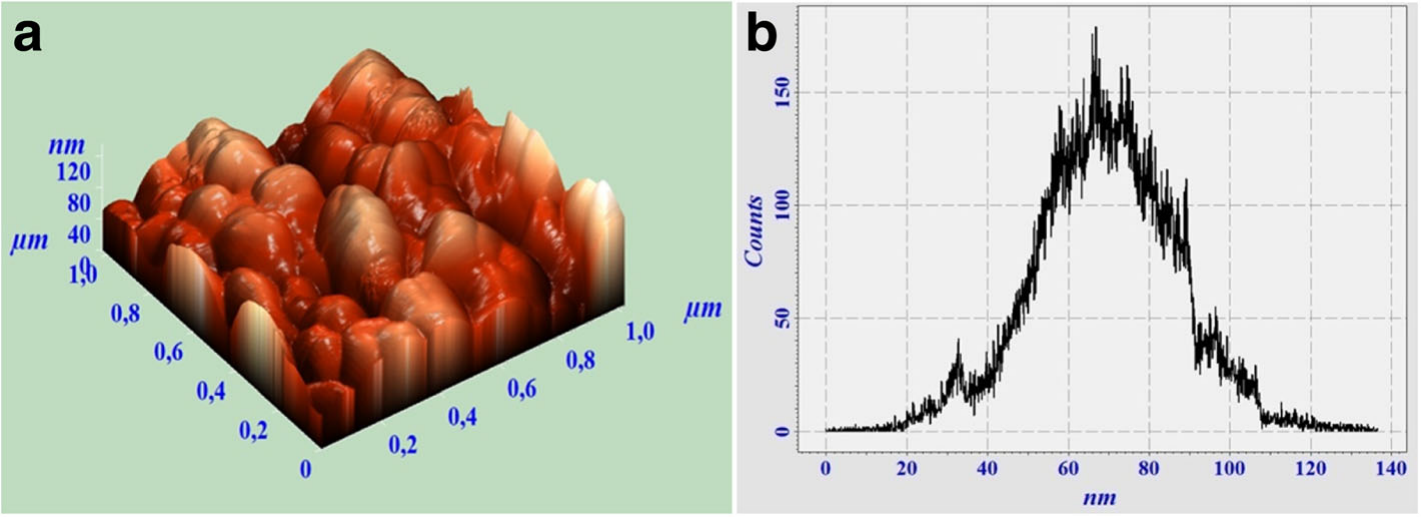
AFM surface image (**a)** and grain-size distribution diagram (**b)** of Gd_3_Ga_5_O_12_ with 1 mol % Tb^3+^ ions synthesized via co-precipitation method with 300 g/mol of polyethylene glycol

**Fig. 4:**
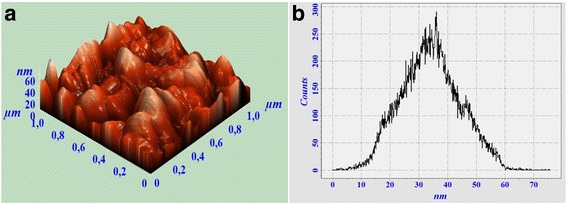
AFM surface image (**a)** and grain-size distribution diagram (**b)** of Gd_3_Ga_5_O_12_ with 1 mol % Tb^3+^ ions synthesized via co-precipitation method with 400 g/mol of polyethylene glycol

Three-dimensional atomic force microscopy (AFM) cross-section analyses of surface and grain-size distribution diagrams of nano-sized gadolinium gallium garnets synthesized via a co-precipitation method with different concentration of polyethylene glycol are shown in Figs. 3 and 4. The surface of the samples are homogeneous and shows narrow grain distributions floating in 45–85 and 25–45-nm ranges for Nano PEG-300 and Nano PEG-400 samples, respectively.

The average grain sizes derived from AFM investigations are 69.2 and 34.1 nm for Nano PEG-300 and PEG-400 samples, respectively. It should be noted, that grain-size distribution diagram of Nano PEG-300 (Fig. 3a) shows deviation from the symmetrical Gauss distribution which can cause an overestimated number of average grain size of this sample.

Thus, estimation of grain size with Scherrer’s equation as well as AFM-calculated average grain size is in good agreement. These results show that the increasing of PEG leads to the decreasing of nanoceramic grain sizes.

**Fig. 5:**
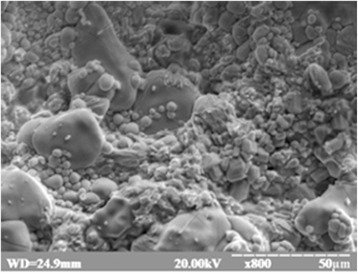
SEM image of cleavage surface of Gd_3_Ga_5_O_12_: 1 mol % Tb^3+^ microcrystalline sample synthesized via solid-state reaction method

Figure 5 shows SEM cleavage surface image of gadolinium gallium garnet microcrystalline sample. The irregular shape of crystallites is observed from this image. It can be seen that microcrystalline sample possesses a broad grain-size distribution. The grain size varies from a few to tens of micrometers. Small-sized grains fill the voids between huge size grains and complete it into the solid ceramic material.

**Fig. 6:**
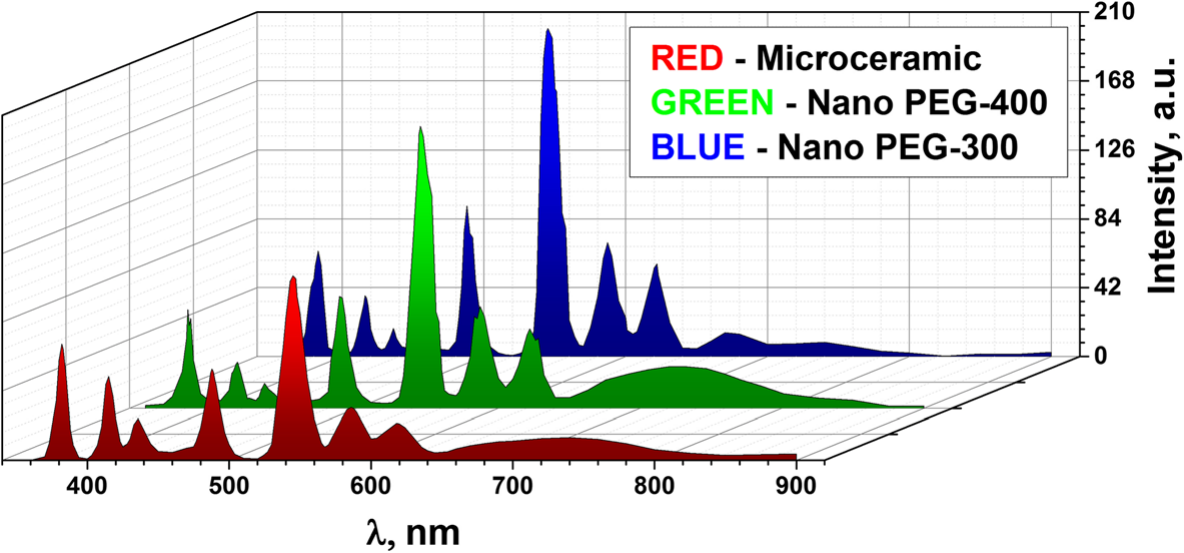
X-ray luminescence of 1 mol % Tb^3+^-doped Gd_3_Ga_5_O_12_ micro- (*red*) and nanoceramics (*green*—Nano PEG-400, *blue*—Nano PEG-300) at room temperature

X-ray luminescence spectra of Tb^3+^-doped Gd_3_Ga_5_O_12_ nano- and microceramics are shown in Fig. 6. Characteristic strong lines assigned to the ^5^D_3_ → ^7^F_*j*_ and ^5^D_4_ → ^7^F_*j*_ (*j* = 6, 5, 4, 3) transitions of the Tb^3+^ ions are observed. These emission lines can be separated in two groups. The blue emission below 480 nm is related with ^5^D_3_ → ^7^F_*j*_ transitions, while the green emission above 480 nm is from ^5^D_4_ → ^7^F_*j*_ transitions. The intensity of the blue emission (Fig. 6) is significantly weaker than the green emission for all ceramics. As it was shown in [9], the spectral energy distribution of Tb^3+^ emission is strongly dependent on the Tb concentration in the case of Y_3-*x*_Al_5_O_12_:Tb_*x*_ powders. The blue emission (^5^D_3_ → ^7^F_j_) dominates for very low Tb concentrations (<0.1%) and completely disappears for concentrations above ~2% [9]. Therefore, when in the investigated micro- and nano-sized samples, the concentration of Tb^3+^ is 1 mol %, the cross-relaxation begins, and the rate for the cross-relaxation depends on the concentration of Tb^3+^ ions [1, 10].

As it can be seen from Fig. 6, the ^5^D_4_ → ^7^F_5_ luminescence transition (green emission) dominates in the X-ray luminescence of Gd_3_Ga_5_O_12_ nano- and microceramics with Tb^3+^-activator concentrations of 1 mol %. There is no significant difference in the shape or band position of the emission lines of the nanocrystalline ceramics with respect to the ceramics prepared by a solid-state reaction method. At the same time, the relative intensity of green emission at 546 nm is higher for Nano PEG-300 and Nano PEG-400 nanoceramics. The ratios of the intensities of the two main emission lines in green (^5^D_4_ → ^7^F_5_ transition) and blue (^5^D_3_ → ^7^F_6_ transition) spectral regions (I_546_/I_384_) are equal to 3.1, 2.8, and 1.6 for Nano PEG-300, Nano PEG-400, and microceramic samples, respectively. Broad luminescence band in the 680–800-nm spectral region is attributed to uncontrolled chromium impurities [11].

It should be noted that the activator emission is enhanced at about 1.5–2 times in the case of nano-sized Gd_3_Ga_5_O_12_: Tb^3+^ ceramics (Nano PEG-300, Nano PEG-400) with respect to the microceramics. The increasing of emission intensity in nanoceramics can be due to the lower content of host defects in the nano-sized materials [8]. Enhanced emission intensity was also observed in the case of Nano PEG-300 sample with respect to Nano PEG-400 sample. Thus, increasing of PEG leads to decreasing of the emission intensity. Also, no appreciable changes in the relative intensity of Tb^3+^ lines were detected in the nanoceramics with PEG-300 and PEG-400.

**Fig. 7:**
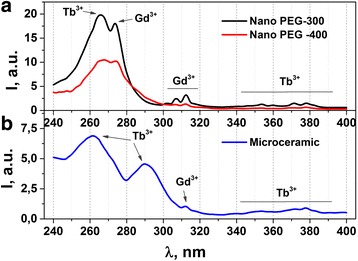
Excitation spectra of nano- (**a)** and microcrystalline (**b)** ceramics of Gd_3_Ga_5_O_12_: 1 mol % Tb^3+^ garnet at 546-nm registration obtained at room temperature

Figure 7 shows excitation spectra of nano- (a) and microcrystalline (b) samples of Gd_3_Ga_5_O_12_: 1 mol % Tb^3+^ garnet obtained at room temperature. Luminescence registration was carried out in the “green” (*λ*_m_ = 546) region of the spectrum. An intense broad band (*λ*_m_ = 266 nm) related to spin-allowed 4*f* ^8^-4*f* ^7^5*d* ^1^ transitions of Tb^3+^ ions [1, 10] and weak lines (*λ*_m_ = 308 and 312 nm) corresponded ^8^S_7/2_ → ^6^P_7/2, 5/2_ transitions of Gd^3+^ ions [3] were found on the photoluminescence excitation spectra of nanocrystalline Gd_3_Ga_5_O_12_: Tb^3+^ ceramics at 300 K (Fig. 7a). Weak lines observed in the spectral region of 350–390 nm at about 354, 360, 372, and 379 nm correspond to ^7^F_6_ → ^5^G_5_, ^5^L_10_, ^5^G_6_, and ^5^D_3_ intracenter transitions of Tb^3+^ ions [9]. At the same time, besides the weak lines attributed to Gd^3+^ and Tb^3+^ ions, also two broad excitation band caused by spin-allowed (*λ*_m_ = 266 nm) and spin-forbidden (*λ*_m_ = 295 nm) 4*f*-5*d* transitions in Tb^3+^ ions are observed in the photoluminescence (PL) excitation spectra of Gd_3_Ga_5_O_12_: Tb^3+^ microceramics (Fig.7b).

**Fig. 8:**
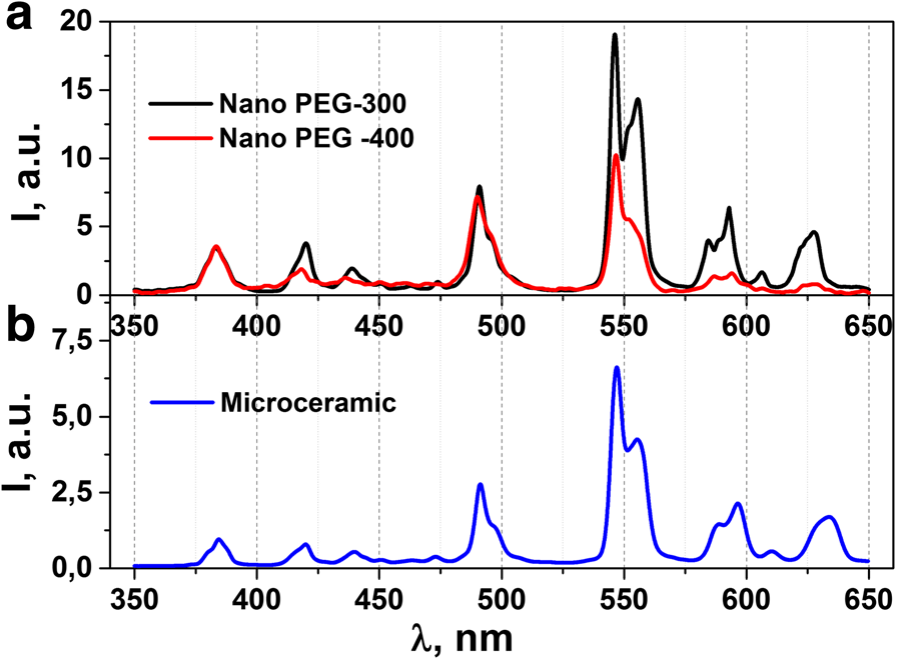
Photoluminescence emission spectra of Gd_3_Ga_5_O_12_: 1 mol % Tb^3+^ nano- (**a**) and microceramics (**b**) at 266-nm excitation obtained at room temperature

Photoluminescence emission spectra of Gd_3_Ga_5_O_12_: 1 mol % Tb^3+^ nano- and microceramic samples obtained at room temperature are shown in Fig. 8. A number of sharp intense lines were found in 350–650-nm spectral regions. These lines correspond to ^5^D_3_ (^5^D_4_) → ^7^F_j_ transitions in Tb^3+^ ions. Sharp weak lines with a maximum at 384, 420, and 440 correspond to ^5^D_3_ → ^7^F_6_, ^5^D_3_ → ^7^F_5_, and ^5^D_3_ → ^7^F_4_ transitions, respectively [1, 9, 10]. It should be noted that samples activated with 1 mol % Tb^3+^ ions reveal relatively high emission in a green spectral region. Luminescence lines in the spectral range of 480–650 nm with maxima at 491 nm (^5^D_4_ → ^7^F_6_), 546 nm (^5^D_4_ → ^7^F_5_), 597 nm (^5^D_4_ → ^7^F_4_), and 633 nm (^5^D_4_ → ^7^F_3_) are observed at longer wavelengths. Emission lines of ^5^D_3_ and ^5^D_4_ levels are associated with weak electron-vibrational transitions in Tb^3+^ ions [1, 10]. When the phosphors were excited with UV radiation of 266 nm wavelength (4*f*-5*d* transition), the Tb^3+^ ion (4*f* ^8^) would be raised to the higher 4*f* ^7^5*d* ^1^ level and would feed afterward to the ^5^D_3_ or ^5^D_4_ excited states [12].

The photoluminescence spectrum of Gd_3_Ga_5_O_12_: Tb^3+^ nanoceramic with PEG-300 is better resolved through higher intensity and more optimal molecular weight of polyethylene glycol. Photoluminescence intensities of investigated micro- and nanoceramics are in good agreement with the X-ray emission intensities.

## Conclusions

The Gd_3_Ga_5_O_12_ nanoceramics doped with 1 mol % Tb^3+^ ions have been successfully obtained via co-precipitation method with aqueous ammonia as precipitant and PEG as polymeric agent. Analysis of the XRD patterns, AFM images, and grain-size distribution diagrams showed that the size of nanocrystallites varies within 25–85 nm. The size of Gd_3_Ga_5_O_12_ garnet nanoparticles decreases with the increasing of PEG concentration. The average grain size of crystallites calculated with Scherrer’s equation is equal to 43.7 and 38.9 nm for Nano PEG-300 and Nano PEG-400 ceramics, respectively. Other concentrations of PEG as well as the different methods of synthesis in Gd_3_Ga_5_O_12_: Tb^3+^ green nanoparticle phosphors still require further research.

The broad excitation band caused by spin-allowed (*λ*_m_ = 266 nm) *4f-5d* transitions of Tb^3+^ ions together with weak lines attributed to Gd^3+^ and Tb^3+^ ions were observed in Gd_3_Ga_5_O_12_: Tb^3+^ nano- and microceramics. Moreover, additional broad excitation band caused by spin-forbidden (*λ*_m_ = 295 nm) *4f-5d* transitions of Tb^3+^ ions is observed in the PL excitation spectra of microceramics.

Strong characteristic lines assigned to the ^5^D_3_ → ^7^F_*j*_ and ^5^D_4_ → ^7^F_*j*_ (*j* = 6, 5, 4, 3) transitions of the Tb^3+^ ions are observed in the luminescence spectra under X-ray and UV excitations. The luminescence ^5^D_4_ → ^7^F_j_ transitions (green emission at 546 nm) dominate in the X-ray and photoluminescence of Gd_3_Ga_5_O_12_ nano- and microceramics with activator concentrations 1 mol % Tb^3+^. There is no significant difference in the shape or band position of the emission curve of the nanocrystalline ceramics Nano PEG-300 and Nano PEG-400 with respect to the ceramics prepared by a solid-state reaction method. No appreciable changes in the relative intensity of Tb^3+^ lines were also detected in the nanoceramics with PEG-300 and PEG-400. Enhanced X-ray and PL emission intensities of the Tb^3+^ ions for the nanocrystalline Gd_3_Ga_5_O_12_ ceramics in comparison with microceramics are due to better stoichiometry and lower content of host defects. Increasing of the PEG from 300–400 g/mol leads to decreasing of the X-ray and PL emission intensities of Gd_3_Ga_5_O_12_ nanoceramics with 1 mol % Tb^3+^ ions.

To conclude, green emission is obtained because the Tb^3+^ concentration is high enough and cross-relaxation from ^5^D_3_ to ^5^D_4_ occurs between adjacent ions. Consequently, it could be suggested that Gd_3_Ga_5_O_12_ nanoparticle phosphors doped with 1 mol % Tb^3+^ ions are promising materials in a green spectral region for different optical applications.

## Abbreviations

AFM: Atomic force microscopy
PEG: Polyethylene glycol
PL: Photoluminescence
PSD: Position-sensitive detector
SEM: Scanning electron microscopy
XRD: X-ray diffraction
